# Linking Measure of the Tropical Stingless Bee (Apidae, Meliponini, and *Heterotrigona itama*) Honey Quality with Hives Distance to the Source of Heavy Metal Pollution in Urban and Industrial Areas in Sabah, Borneo

**DOI:** 10.1155/2022/4478082

**Published:** 2022-09-30

**Authors:** Nurul Hamizah Salman, Lum Mok Sam, Kimberly Ador, Bellericter Binjamin, Mohd Iftar Johwan Johny-Hasbulah, Suzan Benedick

**Affiliations:** Faculty of Sustainable Agriculture, Universiti Malaysia Sabah, Locked Bag No 3, Sandakan 90509, Sabah, Malaysia

## Abstract

Honey is a natural product of bees, and its chemical composition depends on the nectar sources of the surrounding flora as well as environmental factors. However, keeping hives in areas polluted with heavy metals can affect the quality of bee products such as honey. To date, there have been very few studies on the health risks of consuming honey at various locations in the Malaysian state of Sabah, Borneo, in relation to food standards and heavy metal contamination of honey from the stingless bee, *Heterotrigona itama* in association with pollutant sources. A total of 63 samples of raw and unprocessed honey were collected directly from beekeepers producing honey at five sites in the industrial areas. All selected heavy metals were measured using an inductively coupled plasma optical emission spectrophotometer (ICP-OES). Overall, the most frequently detected element was Zn (0.090 mg/kg), followed by Pb (0.012 mg/kg), As (0.004 mg/kg), and Cr (0.003 mg/kg), while Cd (0.001 mg/kg) was the lowest element in honey from all areas. With the exception of Cr and Zn, a significant correlation was found between PCA factor score 1 and heavy metal concentration in honey for Pb, Cd, and As, suggesting that the source of pollution for these metal elements was from hives closer to major roads, cities/town, petrochemical hub, and power plants. Although the heavy metal concentrations in the honey samples did not exceed the food standard limits and therefore do not pose a health risk, the observed increase in heavy metal concentrations in honey in industrial areas could pose a potential risk in the future due to the growing interest in rearing of stingless bees for honey production in these areas of Sabah.

## 1. Introduction

Apiculture and meliponiculture are the most popular honey production practices in tropical countries, and to date, the stingless bee of the species *Heterotrigona itama* is one of the most popular local species for beekeeping in tropical countries such as Malaysia [1]. Honey from stingless bees is now seen as a lucrative new source of income and is rapidly gaining popularity throughout the country, currently fully supported by the Malaysian government. Honey from stingless bees is rich in antioxidant properties as well as antibacterial and antifungal activities [2]. The honey also has a high concentration of polyphenols, flavonoids, and trehalulose sugar compounds, which act as excellent antioxidants, are suitable for diabetics, and can reduce caries complications, which is why this type of honey is sold at higher prices [2]. Honey from stingless bees such as *H. itama* tastes different from honey from honeybees as it has higher water content, lower diastase activity, a different sugar spectrum, and a unique sour taste [3, 4]. All bees collect different types of nectar and pollen from plants to produce honey, which also has different chemical compositions depending on environmental factors.

Honey may not only contain trace metals such as arsenic, cadmium, nickel, chromium, and lead, which are known toxic metals, but also other essential metals such as zinc, copper, and manganese, which are important for human health and development if consumed in a certain amount [5]. However, excessive intake of such elements can be hazardous to human health [6, 7]. Heavy metals such as arsenic, cadmium, chromium, lead, and zinc can hardly be destroyed or degraded [8]. In industrial areas, vehicle exhaust, industrial activities, and weathered materials are the sources of heavy metal pollution. Higher concentrations of heavy metal pollution are usually found in industrial areas than in suburban areas [9, 10]. It is, therefore, important to measure the content of heavy metals in honey to ensure product quality.

Heavy metal toxicity is industrial pollution in areas affected by processes such as factories, power plants, urbanization, major roads, and agriculture. In high concentrations, heavy metals can accumulate in soils and plants, which in turn can be ingested by bees [11]. Many studies have found the accumulation of heavy metal elements in honey due to environmental pollution [6, 7, 9]. Heavy metals present in the atmosphere can be deposited directly on the hairy body of bees or enter the insect via nectar, pollen, honeydew, or water during foraging [12, 13]. The presence of unwanted heavy metal contaminants in honey thus indicates a polluted environment in which the hives or nests are located. Heavy metals and other pollutants in honey from honey bees and stingless bees are the focus of research in a number of tropical and subtropical regions of South America, South Africa, and Southeast Asia, as honey is a good indicator of pollution levels: Venezuela [14], Brazil [15, 16], Indonesia [17], Ethiopia [11, 18], Malaysia [19–24], Bangladesh [25], Nigeria [26], and India [27].

Several recent studies have been conducted to evaluate the chemical composition of Malaysian honey [23, 24]. In a study on the honey of the stingless bee, *H. itama*, the mineral content was investigated in the states of Selangor, Kedah, Johor, Kuala Terengganu, Sarawak, and Sabah [22]. The samples from the forests had higher total K, Sd, Ca, Mg, and Fe content than the samples from the suburban areas, whereas the content of heavy metals (Cd, Sb, Hg, Pb, and As) was less than 0.01 mg/kg. Another study conducted with the same species in the Malaysian state of Sarawak, comparing their honey with honey from Brazil, New Zealand, and China, showed that the average concentration of Zn, Cu, and Pb was much lower, while As and Cd below the detection limit [23]. However, all the studies published so far do not describe a comprehensive analysis of the heavy metal composition of honey from different locations, especially near urban and industrial areas such as this study. Furthermore, little is known to date about the extent to which the location of hives near urban and industrial areas in the state of Sabah, Malaysia, may influence the accumulation of heavy metals in stingless bee honey and the associated potential risks to human health.

The aim of this study was to determine the levels of heavy metals (arsenic (As), cadmium (Cd), chromium (Cr), lead (Pb), and zinc (Zn) in the honey of stingless bees (*H. itama*) collected from beekeepers in urban and industrial areas. Recently, there has been a growing interest among beekeepers to raise stingless bees in urban and industrial areas in the Malaysian state of Sabah. Thus, this study will help to ensure the safe consumption of honey while providing beekeepers with additional information on the importance of keeping stingless bees in a place where the risk of heavy metal contamination to bees and human health is low.

## 2. Materials and Methods

### 2.1. Sample Collection Area

**Figure 1:**
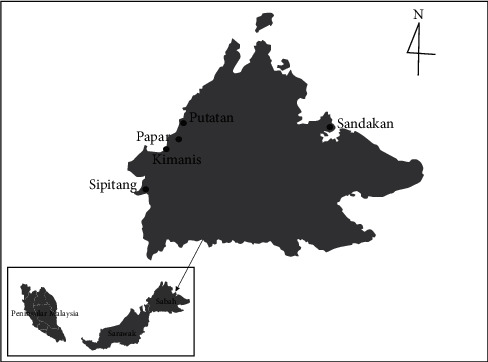
Localities of honey sampling in the State of Sabah, Malaysia.

A total of sixty-three samples of honey from the stingless bee *H. itama* were collected fresh and unprocessed from the beehives of local beekeepers in the districts of Sandakan, Sipitang, Papar, Kimanis, and Putatan in Sabah, Malaysia, from 2019 to 2021 (Figure 1; Table 1).

### 2.2. Method of Analysis

The dry ash method was used for the extraction of heavy metal elements in honey samples by ICP-OES. Approximately 5.0 g of honey was placed in a crucible and dried in an oven at 100°C for 24 hours before being burnt to ash in an oven at 550°C for 9 hours the next day. The resulting white ash was then diluted in 5% (w/v) nitric acid and made up to 50 mL with distilled water (Elga Option-R 15 PURELAB Water Purification System). Samples were extracted twice to completely remove small precipitates and analyzed using the Perkin Elmer 5300 DV ICP-OES. The measuring wavelengths of the measured elements in the ICP-OES were as follows: As (396.152 nm), Cd (228.802 nm), Cr (267.716 nm), Pb (220.353 nm), and Zn (206.200 nm) [24]. Triplicate honey samples were prepared for analysis along with blanks and standard solutions to reduce error. Distilled water (Elga Option-R 15 PURELAB Water Purification System), Baker Analyzed reagent HNO3 69%, 70%, multielement Calibration Standard 3, matrix per volume: 5% HNO3 per 125 ml, and the OPTIMA Blank Solution, 2% HNO3, 500 ml were used. All chemicals and reagents used were of analytical grade.

### 2.3. Abiotic Factors and Distance to Industrial Areas

Climate data were collected at each site during fieldwork in January and February 2019. The inventory of environmental variables measured temperature (°C) and relative humidity (RH), as well as distances of the hive from industrial areas, including distance from the petrochemical hub (km), distance of the site from the power plant (km), distance of the site from the main road (km), and distance of the site from the nearest town (km).

### 2.4. Health Risk and General Standard for Heavy Metals in Foodstuffs

To assess the contamination of honey with heavy metals from the industrial area, the levels of As, Pb, and Cd found in this study were compared with the maximum permitted proportion of metal in honey, according to the Codex Alimentarius Commission [28, 29] and Malaysian Food Regulations [30]. The maximum permitted proportion of metals in honey for As, Pb, and Cd were 1 mg/kg, 2 mg/kg, and 1 mg/kg, respectively. For Cr and Zn, there is currently no health reference standard for honey in Malaysia, so the risk assessment was based on the international food standard. The Agency for Toxic Substances and Disease Registry (ATSDR) recommended mean daily intake in food for healthy adults is estimated to be 12–15 mg/day for Zn [31, 32] and 0.02–0.045 mg/day for Cr [31].

### 2.5. Statistical Analysis

All data were expressed as mean ± standard error (SE) of triplicate samples. Significant differences in heavy metal concentrations in honey from all study areas were determined using ANOVA and Tukey's *b*-test (*P* < 0.05). Abiotic factors and environmental variables were analyzed using principal component analysis (PCA). Pearson's correlation analysis was also performed to determine the correlation between heavy metals, abiotic factors, and distance of the hive from the pollution source.

## 3. Results and Discussion

### 3.1. Determination of Selected Heavy Metals in Stingless Bee Honey

The determination of heavy metals in honey samples is presented in Table 2 in the statistical data of mean total concentration ± SE of selected heavy metals (As, Cd, Cr, Pb, and Zn) in *H. itama* samples from five areas of Sabah. Zn was found most frequently in the honey of stingless bees of Sabah (0.090 mg/kg), followed by Pb (0.012 mg/kg), As (0.004 mg/kg), Cr (0.003 mg/kg), and Cd (0.001 mg/kg) (Table 2). The zero value of the mean As and Cd concentration of nine honey samples in Sandakan was due to the concentration of the detected element being too low and below the limit of quantification of the analytical method ICP-OES. Based on the Codex Alimentarius Commission [28, 29] and Malaysian Food Regulations [30], the range of mean metal concentrations in honey samples from all study areas was relatively low for As (<0.000 to 0.008 mg/kg), Cd (<0.000 to 0.002 mg/kg), and Pb (0.005 to 0.021 mg/kg) (Table 2). In this study, the range of mean Zn (0.059 to 0.106 mg/kg) and Cr (0.001 to 0.002 mg/kg) concentrations in honey was also below the recommended daily food intake for healthy adults, according to the Agency for Toxic Substances and Disease Registry (ATSDR) (Table 2) [31, 32]. It is necessary to determine the concentrations in honey that are acceptable for health because exceeding the bioaccumulation of contaminated honey may lead to increased intake of toxic elements. Honey of high quality and safety will protect public health.

Zn is crucial for important biological processes in bees, such as nutrient metabolism and cuticle development [33], which probably explains the relatively high concentration of Zn in all honey samples compared to other metals (Table 2). Zn is a common heavy metal in honey from stingless bees, which has been confirmed by other studies in Malaysia [1, 20–23], Brazil [15, 16], Nigeria [26], and Indonesia [3, 17]. Although Zn is an essential element for the human body, high Zn intake can lead to adverse health effects. Therefore, quality control of honey is necessary. In this study, the range of mean Zn concentration in meliponine honey (Table 2; 0.059 to 0.134 mg/kg) was much lower than in honey in other countries such as Brazil (0.4000 to 0.9278 mg/kg) [16] and Nigeria (1.756 to 3.992 mg/kg) [26]. Compared to other states in Malaysia, the overall mean Zn concentration in honey from urban and industrial areas in Sabah (0.090 mg/kg) was also lower than that of honey samples collected from Peninsular Malaysia in suburban Kuala Nerang, Kedah (1.16 mg/kg), University of Putra Malaysia, Selangor (2.21 mg/kg), Muar, Johor (2.11 mg/kg) Kuala Terengganu, Terengganu (3.03 mg/kg), and Tumpat, Kelantan (1.67 mg/kg) [22]. In addition, honey samples found in the jungle and secondary forests of Negeri Sembilan, Johor, and Pahang also had higher Zn contents, ranging from 1.76 to 4.25 mg/kg [1].

Pb, As, and Cr are nonessential toxic metals but naturally occurring elements in the environment that can enter the human health system through food, water, and air [31]. These heavy metals have no useful role in human metabolism and can cause health disorders as toxicity progresses even in trace amounts [31]. Pb, As, and Cd do not degrade in the environment [31], and in this context, the anthropogenic source of contamination with these metals in the honey samples was probably urban development, transport, and industrial activities (Table 1). Small amounts of Cr occur naturally in a variety of foods such as fruits, vegetables, beverages, and meat [31]. Cr present in studied honey samples was also could be due to contact with stainless-steel surfaces during harvesting and processing, as honey is corrosive due to its acidity [34, 35]. It is, therefore, important that local beekeepers are advised to avoid the use of stainless-steel tools, especially when handling bee products. Compared to honey from stingless bees in this study, the average concentration of heavy metals in honey in Nigeria was lower, i.e., Pb was not detected in five out of six samples (<0.000 to 1.000 mg/kg), Cr (0.0019 to 0.0297 mg/kg), and Cd (<0.001 mg/kg) [26]. In Brazil, the mean concentration of Pb and Cd in the honey of the stingless bee, *M. scutellaris,* was relatively the same as in this study, ranging from <0.000 to 0.0007 mg/kg and 0.000 mg/kg to 0.0010 mg/kg, respectively, but Cr was much higher, ranging from 0.2806 to 0.5513 mg/kg [16].

Bornean honey from *H. itama* has been reported to be of high quality and contains much less or no heavy metals compared to other bee species, e.g., *Apis* spp. [17, 23]. In the study in East Kalimantan, Indonesia, it was found that Pb, Cd, and As were not detected in most honey samples from *H. itama* [17]. In another study with honey from Malaysian Borneo in Sarawak State, lower mean concentrations of Pb (0.010–0.012 mg/kg) and no detections of As and Cd were also found [23]. In this study, the mean concentrations of Pb (0.005 to 0.021 mg/kg), As (0.000 to 0.008 mg/kg), Cd (0.000 to 0.002 mg/kg), and Cr (0.000 to 0.003 mg/kg) in Bornean honey of *H. itama* were consistent with the above results, which were far below the recommended levels (Table 2). However, in the studies of several states in Peninsular Malaysia (Kedah, Selangor, Johor, Kuala Terengganu, and Kelantan), the mean concentration of As, Cd, and Pb in honey of the same species was slightly higher, about 0.01 mg/kg for Cd and As and about 0.10 for Pb [22] (Table 2). Another study on the mean concentration of Cr in honey of *H. itama* in southern Negeri Sembilan, northern Johor, and western Pahang of Peninsular Malaysia was also much higher (0.27 to 0.82 mg/kg) [1]. Overall, our data conclude that the quality of honey in terms of heavy metal content is satisfactory and that there is no concern for heavy metal toxicity after consumption of Bornean honey from the Malaysian state of Sabah.

### 3.2. Determinants of the Heavy Metals Contamination in Honey in Relation to the Distance of the Hive from Sources of Industrial Pollution and Selected Abiotic Factors

Two components of variation were extracted from the six variables using principal component analysis (PCA), which explained a weighting of 85.15% of the variability in the data sets. PCA factor score 1 explained more than 56.07% of the variance in the data sets and increased in order of importance with increasing distance of hives from the type of vegetation, distance of hives from the power plant, distance of hives from the petrochemical hub, distance of hives from the main road, and distance of hives from the city/town (Table 3). PCA factor score 2 explained more than 29.08% of the variance in the data set and increased in order of importance with increasing relative humidity and temperature (Table 3). With the exception of Cr and Zn, a significant relationship measured by Pearson's correlation was found between PCA factor score 1 and heavy metals in honey for As (*P* < 0.001), Cd (*P*=0.01), and Pb (*P*=0.05), suggesting that the accumulation of these three toxic metals in the honey samples was related to the location of the hives in the area where they are produced. This was also confirmed by the fact that areas closer to the main road, city, petrochemical hub, and power plant (Sipitang, Kimanis, and Papar) generally had higher levels of As, Cd, and Pb in the honey of stingless bees than areas farther away from these sources of pollution (Putatan and Sandakan) as measured by one-way ANOVA (Table 1; *P* < 0.01). There were no other significant correlations between factor 2 and all types of heavy metals content in honey (*P* > 0.05), indicating that the source of honey pollution was not due to abiotic factors. Although our data showed that heavy metal levels in honey were relatively low compared to the food limits set by the Codex Alimentarius Commission (FAO/WHO), the Malaysian Food Regulations 1985, and the Agency for Toxic Substances and Disease Registry (ATSDR), there was a trend of elevated As, Cd, Cr, and Pb levels in Sipitang, Papar, and Kimanis, which are all located near major roads, cities, petrochemical hub, and power plants (Tables 1 and 2). The studies on the honey of Romanian honey bee showed that elevated levels of heavy metals were found in some of the samples from industrial areas and Cd and Pb exceeded the maximum permissible levels in some samples [36]. A recent study in Lithuania indicated that the concentration of heavy metals (Cd, Cr, Cu, Pb, and Ni) in honey samples were found to significantly decrease with increasing distance from potential pollution sources [7]. There was a possibility that the accumulation of Pb and Cd in this study was caused by car tyres, engine oil consumption, brake abrasion, and road surfaces, which in turn causes these metals to accumulate in the soil before they are taken up by plants through the roots, as has been found in Jengka, Malaysia [37] and may therefore be present in honey. As, on the other hand, can accumulate in soils and plants due to pollution from factories and the intensive use of insecticides and agrochemical fertilizers [38]. Concentrations were relatively high in Sipitang, Papar, and Kimanis, as all these areas are close to agricultural farm in addition to factories and urban areas. The main anthropogenic sources of Zn in the environment (air, water, and soil) are mining and metallurgy with Zn as well as the use of products containing zinc, e.g., agrochemicals [31]. The relatively high distribution of Zn in Sandakan (0.134 mg/kg) compared to the other areas was probably related to soils polluted with Zn, where Zn is used as essential micronutrient in agricultural farm such as oil palm plantation. The growth of oil palm in Malaysia is highly dependent on the availability of nutrients such as Zn supplied through fertilizers [39]. A lack of Zn has been shown to inhibit the growth of oil palm plants and roots [39]. The second highest average Zn concentration in honey (0.106 mg/kg) in Sipitang could also be related to nonmetallic mineral mining and quarrying, which is the main activity in the area. In Nigeria, the Zn content in honey samples was highest when the hives were located near a farm [26]. This finding, therefore, underlines the importance for beekeepers to choose suitable locations for hives that are less contaminated with heavy metals by assessing the distance to sources of pollution in the surrounding area.

## 4. Conclusion

In this study, a total of 63 honey samples of the stingless bee, *H. itama*, were collected from 5 different sampling sites in Malaysian Borneo (Sabah). The samples represented potentially contaminated sites with various pollution sources such as major roads, cities, petrochemical hubs, and power plants. According to the results, the honey samples from Borneo have very low heavy metal levels and are within the safe food limits set by the Codex Commission (FAO/WHO), the Malaysian Food Regulations 1985, and the Agency for Toxic Substances and Disease Registry (ATSDR). However, exposure of honey to heavy metals may be increased if hives are located near polluted industrial areas. In this study, a significant negative correlation was found between Pb, As, and Cd in honey and PCA factor score 1 (*P* < 0.05) with increasing distance from power plant, petrochemical hub, main road, and city. Pb, Cd, Cr, and As are also included in the list of toxic substances published by the Agency for Toxic Substances and Disease Registry, and in this study, these metals increase in the honey samples with decreasing distance from the pollution sources. To ensure that the quality of Bornean honey is maintained and is safe for consumption, stingless bee beekeepers should be mindful of where they place their hives, as bees may forage for floral or nonfloral resources that have high concentrations of heavy metals in the environment.

## Figures and Tables

**Table 1:** Location of honey sampling sites by distance from contamination sources in the Malaysian state of Sabah.

Sampling sites	Total honey samples (*N*)	Industrial area (range of distances)			
		Main road (km)	Nearest cities/town (km)	Petrochemical hub (km)	Power plant (km)
Sipitang	15	0.02 to 0.50	2.92 to 10.93	1.43 to 12.34	50 to 5.02
Papar	12	0.30 to 4.13	0.01 to 4.13	12.06 to 13.60	9 to 9.15
Kimanis	15	0.05 to 0.73	2.69 to 7.84	0.55 to 6.90	0.40 to 4.8
Putatan	12	0.26 to 1.80	0.64 to 2.89	47.11 to 49.36	29.9 to 30.01
Sandakan	9	5.0 to 5.5	15.2 to 15.5	399 to 401	379 to 380

**Table 2:** Mean concentrations of heavy metals in honey (SE) from five sites.

Sampling sites	Heavy metals in honey samples mean ± SE (mg/kg)				
	As	Cd	Cr	Pb	Zn
Sipitang	0.008^c^ ± 0.001	0.002^b^ ± 0.000	0.003^bc^ ± 0.000	0.021^b^ ± 0.014	0.106^ab^ ± 0.013
Papar	0.007^c^ ± 0.002	0.001^ab^ ± 0.000	0.003^c^ ± 0.000	0.007^ab^ ± 0.001	0.073^ab^ ± 0.011
Kimanis	0.005^bc^ ± 0.001	0.002^b^ ± 0.000	0.002^b^ ± 0.000	0.018^ab^ ± 0.005	0.077^ab^ ± 0.013
Putatan	0.003^b^ ± 0.001	0.001^ab^ ± 0.000	0.001^ab^ ± 0.000	0.007^ab^ ± 0.003	0.059^a^ ± 0.024
Sandakan	<0.000^a^ ± 0.001	<0.000^a^ ± 0.000	0.002^a^ ± 0.000	0.005^a^ ± 0.000	0.134^b^ ± 0.013
Mean total	**0.004** **±** **0.001**	**0.001** **±** **0.000**	**0.003** **±** **0.000**	**0.012** **±** **0.003**	**0.090** **±** **0.014**

Mean ± standard error (SE) values in the same column with different superscript letters are significantly different (P < 0.05). As, Arsenic; Cd, Cadmium; Cr, Chromium; Pb, Lead; Zn, Zinc.

**Table 3:** Contribution of different environmental variables to the two factor scores in the PCA analysis.

Variable	Weighting	
	Factor 1	Factor 2
Type of vegetation	**0.61**	0.55
Temperature	0.46	**0.85**
Relative hHumidity	−0.033	**0.85**
Distance of hives from the main road	**0.78**	0.41
Distance of hives from the factory	**0.97**	0.21
Distance of hives from the coal power plant	**0.99**	0.06
Distance of hives from the town	**0.84**	−0.06

^*∗*^ *Note*. Variables contributing most to each principal component (>0.60) are highlighted in bold.

## Data Availability

The data supporting the findings of this study are included in the manuscript.
